# Involving Users to Improve the Collaborative Logical Framework

**DOI:** 10.1155/2014/893525

**Published:** 2014-01-23

**Authors:** Olga C. Santos, Jesus G. Boticario

**Affiliations:** aDeNu Research Group, Computer Science School, UNED, Calle Juan del Rosal 16, 28040 Madrid, Spain

## Abstract

In order to support collaboration in web-based learning, there is a need for an intelligent support that facilitates its management during the design, development, and analysis of the collaborative learning experience and supports both students and instructors. At aDeNu research group we have proposed the Collaborative Logical Framework (CLF) to create effective scenarios that support learning through interaction, exploration, discussion, and collaborative knowledge construction. This approach draws on artificial intelligence techniques to support and foster an effective involvement of students to collaborate. At the same time, the instructors' workload is reduced as some of their tasks—especially those related to the monitoring of the students behavior—are automated. After introducing the CLF approach, in this paper, we present two formative evaluations with users carried out to improve the design of this collaborative tool and thus enrich the personalized support provided. In the first one, we analyze, following the layered evaluation approach, the results of an observational study with 56 participants. In the second one, we tested the infrastructure to gather emotional data when carrying out another observational study with 17 participants.

## 1. Introduction

Our experience shows that providing cooperation services as forums, chats, or shared file storage areas does not mean that the learners will work in collaboration. As reported in [1], students who participated in a well-known activity for workgroups called the Logical Framework Approach [2] complained of the lack of collaboration as the only way to collaborate was through messages on a forum. As a result of these findings, we proposed a collaborative extension to the Logical Framework Approach called the Collaborative Logical Framework (CLF) [1]. The CLF is a domain-independent web-based collaborative tool supported by adaptive tasks, which draw on user modeling by data mining learners' interactions. It has been implemented [3] in a well-known open source learning management system called OpenACS/dotLRN [4].

The CLF main objectives are creating effective scenarios that enable learning through interaction, exploration, discussion, and collaborative knowledge construction. To this, the approach takes advantage of advanced information and communication technologies supported by artificial intelligence techniques to allow students to collaborate anytime and anywhere. It follows the Web 2.0 philosophy as the participation of the students is fostered in the process and supported in the authoring of their own contributions in a collaborative way.

Moreover, the design of the CLF, as in any other effective web collaborative learning environment, must primarily ensure that collaborative learning takes place. This cannot be confirmed merely by offering a set of communication tools and a set of collaborative tasks to students in these environments [5]. These authors establish five conditions, which have been followed in designing the CLF, to make collaborative learning better than individual or competitive learning: (1) positive interdependence (everyone shares the goals), (2) individual accountability/personal responsibility (everyone is in charge of oneself), (3) promoting interaction, (4) interpersonal and small group skills (everyone works effectively with each other and functions as part of a group), and (5) frequent and regular processing of the group's functioning to improve its effectiveness in the future should all be clearly perceived. The first four conditions are related to the collaborative task design, while the fifth one is especially suited for providing some support from the learning environment, as the CLF does.

Consequently, in order to support collaboration for both students and instructors in web-based learning, there is a need for an intelligent support that facilitates its management during the design, development, and analysis of the collaborative learning experience. The management of collaborative learning interaction can be divided into three categories: (i) mirroring systems, which display basic actions to collaborators, (ii) metacognitive tools, which represent the state of interaction via a set of key indicators, and (iii) coaching systems, which offer advice based on an interpretation of those indicators [6].

In e-learning environments the interaction analysis is usually not an easy task due to the large number of students and, therefore, the high number of interactions. Thus, when designing a collaborative learning environment, an intelligent support has to be provided to analyze the student collaboration regularly and frequently with little intervention by the instructor. In this way, the instructors' workload can be reduced as some of their tasks—especially those related to the monitoring of the students behavior—are automated. If possible, this intelligent support should be provided in a domain-independent way to facilitate transferability and analysis without human intervention [7].

In previous aDeNu work [7], domain-independent statistical indicators of students' interactions in forums (i.e., conversations started, messages sent, and replies to student interactions) were identified by mining non-scripted collaborative interactions. Benefits of their awareness by students were evaluated. In the same way, the CLF can enrich student's metacognitive support by adding automatically inferred indicators from student's interactions [8]. In this context, a review of the literature of intelligent systems for collaborative learning support [9] agrees that data mining techniques can be applied to support an automated process of analysis, while a rule-based approach can be used to deliver adaptation to the learners. The latter can also promote effective collaborative learning while accomplishing the task [10].

Given that emotions can emerge in collaboration scenarios [11] and can be very motivating and rewarding for learners [12], socioemotional states can also be considered to overcome problems encountered by distant collaborators [9]. Data mining techniques are also useful for emotional information detection in collaborative scenarios [13].

All the above should be taken into account when designing the collaboration tool. However, designing the collaboration support is not sufficient. Formative evaluations are also needed to assess that the system developed truly meets the user requirements [14].

Bearing all this in mind, the objectives of our work through the usage of the CLF have been as follows: (1) supporting design, development, and analysis of effective scenarios that enable learning through interaction, exploration, discussion, and collaborative knowledge construction, (2) providing intelligent support to analyze the student collaboration regularly and frequently with little instructor's intervention, (3) assessing collaborative learning through domain-independent statistical indicators inferred by data mining techniques and following the layered evaluation approach, and (4) evaluating the effectiveness of the approach. Further we are currently involved in extending the CLF to manage emotional indicators and personality traits.

In particular, in this paper we recap the CLF approach and discuss two formative evaluations that we have carried out to improve the design of this collaborative tool and thus enrich the personalized support provided during the collaboration. To this, after reviewing the research background on the collaboration issues related to our work, we present the relevant aspects of the CLF to support the collaboration among students. Then we describe two observation studies carried out with users to improve the design of the CLF. After that, we discuss the implications of the outcomes of these studies for the design of forthcoming experience. We end with some conclusions.

## 2. Background

There is vast research arguing on the advantages of using collaborative learning, and significant efforts have been made on characterizing the main issues involved [5, 6, 15, 16]. To take advantage of these developments, it is advisable to bear in mind that the ultimate goals are twofold. On the one hand, collaborative learning aimed at actively engaging students in the learning process through social interactions with their mates so that overlapping backgrounds and knowledge complement a particular student's concepts and understanding. This goal draws on well-known Vygotsky's theories on social connections [17, 18]. On the other hand, there are benefits related to the achievement of teamwork skills and functional knowledge [19], where team member interacts with another so as to facilitate the progress of the team as a whole [20].

However, developing successful collaborative environments is not trivial and several conditions have been identified to make collaborative learning better than individual or competitive learning [5]. The aforementioned five conditions pose critical issues when facing real situations that are commonly taking place in current web-based collaborative environments. The problems involved are not new and relate to the very characterization of collaborative learning, which relies on several factors such as (1) mutual interdependence on students' behavior and how different roles (such as coordinators, moderators, and managers) could facilitate collaboration progress [21], (2) required structuring of the learning task [22], (3) desired complementary features of students' profiles while establishing group members [23], (4) variety of source data and inference methods involved [24], (5) difficulties involved in evaluating collaborative learning processes [25], (6) self-regulation role in managing social and collaborative activities [26], and especially (7) lack of consensus on how to model collaboration [27]. A major conclusion from all these issues is that there is lack of methodology and standards to analyze collaboration [28].

Managing the collaboration in e-learning scenarios requires several issues,as discussed in [8]. First is characterizing the collaborative experience. This implies modeling the collaboration with a set of indicators. These indicators can be of diverse nature, such as number of system accesses per student, number and mean of contributions made, kind of the contributions, and depth of the discussions [29], or user behavior indicators that indicate most active users, relevant users, and so forth [30].

Second is supporting monitoring and analysis of interactions. Here machine learning techniques have been used to analyze student collaboration and grouping of students according to their collaboration using unsupervised classification techniques [31].

Third is identifying data sources and data acquisition models. With respect to data sources, user modeling considers user, usage, and environment data [32]. A typical source of data in collaboration scenarios is interactions in forums as they can be used to infer the level of activity or frequency of use [33]. In turn, data acquisition methods can be of diverse nature: (a) qualitative, where the individuals participating in the research are directly questioned or experts assess participants' activities; (b) quantitative, where statistical information on participants' activities is collected; and (c) mixed, where both methods are used simultaneously.

Fourth is providing appropriate inference methods to support adaptive tasks. In this case, inference methods can be diverse, and consider expert's analysis [34], comparison with a preexisting model [29], interaction analysis, mainly statistically oriented [25, 30, 35, 36], and machine learning techniques [29, 31, 37], among others.

Fifth is encouraging the consideration of self-regulated features, such as metacognitive skills, which can help students and improve their learning [38] and which involve motivational and emotional aspects [39]. In this context, open learner models [40] can be used to show and manage the results of the collaboration analysis in order to increase self-learning awareness.

Sixth and last thing is validating the effectiveness of the collaboration through evaluation. The evaluation of intelligent collaborative learning environments as any other adaptive systems evaluation is still an open field [14]. It can be addressed from different perspectives. One of them is the layered evaluation approach. Here, the adaptation process is broken down into its constituents—called layers—and each of these layers is evaluated separately where it is necessary and feasible to learn about what causes success or failure in the adaptive response. The most up-to-date framework is the one proposed by Paramythis et al. [41]. It defines five layers, corresponding to the main stages of adaptation: (1) collection of input data, (2) interpretation of the collected data, (3) modeling of the current state of the world, (4) deciding upon adaptation, and (5) applying adaptation. Another approach comes from the usability field and involves observing users carrying out the tasks to understand their behavior [42]. In particular, observational studies allow viewing what users actually do in context. These studies can combine direct observation, to focus attention on specific areas of interest, and indirect observation, to capture activity that would otherwise have gone unrecorded or unnoticed.

As anticipated in the introduction, emotions are inherent in collaboration scenarios, and thus, they have to be considered when managing the collaboration in e-learning scenarios. However, up to our knowledge, little attention has been paid on understanding the role of affective and social factors when learning collaboratively and there is still a need for further development of methodological approaches [12]. Some studies have used the Self-Assessment-Manikins (SAM) [43], a nonverbal instrument measuring two distinct dimensions of emotions (valence and arousal) by means of graphic representations of mood in the form of manikins, based on the circumplex model of affect [44] to evaluate the influence of an emotion representation tool in different collaborative work environments [45]. Moreover, personality traits and emotions also play a key role in social and collaborative scenarios [46]. In this sense, personality can modulate the way the student participates in a given situation. For instance, some studies have found that participants that exhibit lower scores on extraversion and higher ones on mental openness prefer online learning [47].

All the above considerations have implications in the management of the collaboration. From the practical viewpoint, previous to the beginning of the collaborative experience it is necessary to design carefully the activity, taking into account the different aspects that might structure the learning experience, such as the context, the group size, the group composition, the collaborative task, or the definition and distribution of the participants' roles [15]. The latter is of particular importance, since interaction patterns depend on the roles assumed by participants in the learning process, and teachers need support to be able to detect these emergent roles and undesired interaction patterns [48]. It is well known that a key figure for achieving effective learning is the group administrator or moderator [21]. As we have discussed elsewhere [49], roles can elicit additional emotional reaction or modulate existing ones.

## 3. The Collaborative Logical Framework

We proposed a collaborative extension of the Logical Framework Approach called the Collaborative Logical Framework (CLF) to better support students in collaborative learning [1] and allow for real collaboration among them by making students work consecutively in three ways: (i) answering the questions individually, (ii) working in cooperation with their colleagues' answers, and (iii) working all together to reach an agreement. The support provided covers two objectives: (1) encourage students to work in groups, and (2) train students in reaching an agreement when solving problems.

**Figure 1:**
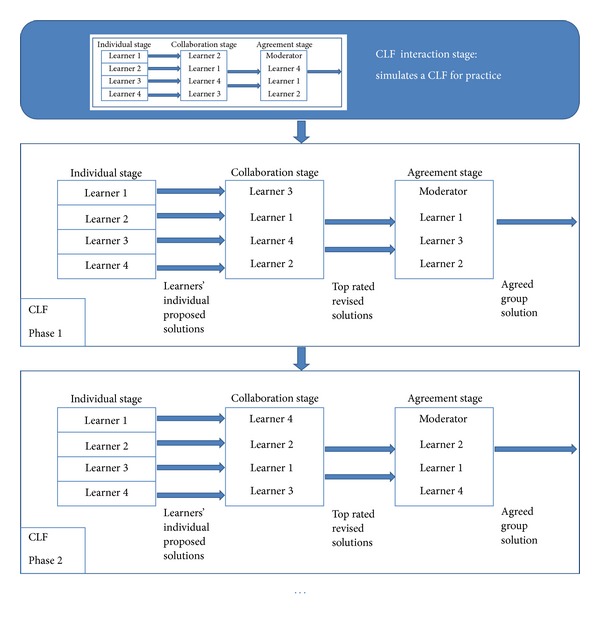
CLF structure.

The CLF is designed as a domain-independent tool and is supported by a user model built from learners' interactions within the collaboration task. The goal is that learners work collaboratively to provide an agreed solution. An interaction stage can take place once at the beginning of the CLF. This stage is preparatory and simulates a CLF to teach the CLF methodology and facilitate the deployment of the CLF by gathering interaction data that can be used to build an initial collaboration model of the learners. After that, several CLF can be carried out, as outlined in Figure 1.

To facilitate an efficient collaboration, learners are divided into subgroups and, at some point in time, one learner of each subgroup is chosen as its moderator to promote collaboration and communication inside it. Both tasks—automatic creation of subgroups and choosing the moderator—can be done automatically by the system based on the learners' user model. Although the number of participants per group is not predefined, groups in collaborative learning usually have a reduced number of members [5], with four being the number we have used.

The CLF consists of one or several identical phases, where the following three stages are defined. The involvement in each of them is controlled by the different roles learners have during the interaction. For each of the phases, the following stages take place.

- *Individual Stage*. Each learner works individually to produce her contribution on a given problem. When finished, she must start a thread in the forum justifying the solution produced. During this stage, learners can solve their doubts posting messages in a general forum.
- *Collaboration Stage*. Learners have access to the solutions of their mates and must comment—by answering the corresponding forum thread—and rate them (passive collaboration). Once they have analyzed the work of the other learners, each learner has to create a new version of her own work taking into account the comments and ratings given to her by her mates and start a new thread in the forum (active collaboration). Learners can also reject their new version if they are rated lower than the previous one. As a result, the other mates receive a notification of the new version and have to comment and rate it, as before. In any case, discussions take place in the corresponding thread.
- *Agreement Stage*. Taking into account the interactions in the two previous stages of the current phase, a moderator is selected for the group. The moderator of the group is responsible for providing the agreed solution of the group. She has to propose a solution based on the best rated works of the group and make it available to the group members. The procedure is similar to the one described in the previous stage. The moderator produces solutions and justifies them in a new thread. The rest of the members of the group have to rate and comment on the given thread. This stage ends when the task deadline arrives.

In order to describe the management of the intelligent support, as implemented in the CLF [3], we follow the distinctions suggested elsewhere [24] and analyze thus the source of data used (data acquisition), the inference method (modeling the collaborative behavior), and the adaptation processes. After that, some remarks on the CLF are reported.

### 3.1. Data Acquisition

Data acquisition is done by tracking both passive and active students' interactions [50]. Not all the interactions that take place in the system are relevant to the intelligent support required, but only those related to the metrics are defined in the following Section 3.2.

The metrics gather information on user's actions, but regarding the purpose of those actions, we can also characterize them as active or passive. The former refers to actions that denote object creation or data taken directly from database depicted as an absolute value: total of students taking part in a forum, number of messages sent in a thread, number of replies in the own forum, the best rating received, number of versions created, average rating of the live version, and so forth. The latter focuses on the visits to specific pages of the course: average of pages visited in a session, number of messages in the forum before creating a new version, what the student does before creating a version, times when the learner changes her ratings, what the student does after reading the messages in other forums, and so forth. In order to manage them, for each task in the CLF, the system stores all the actions carried out before and all the actions carried out afterwards, including the number of times each action is carried out.

### 3.2. Modeling the Collaborative Behavior

The data acquired is used to model the collaborative behavior of the learners. The CLF gathers the students' performance to know how they work in the course. By means of a relatively wide range of domain-independent metrics the system derives their behavior related to the collective task, focusing the analysis on the forum participation, on the ratings they give to their colleagues' contributions, on the solution versions they create, and on studying actions they carry out before and after a specific operation.

On the one hand, this information is used to get the collaboration indicators, which cover the definition of the learner's reputation. On the other hand, it helps the student and the instructor to monitor the tasks and support the scrutability of the model.

Twelve collaboration indicators have been proposed [51] but are to be further refined from formative evaluations such as the ones reported in this paper. Six of them are active (communicative, insightful, not collaborative, participative, and useful) and the others are passive (gossip, inspirator, inspirable, unsecured, thinker-out, and thorough). The former measure actions related to object creation, while the latter look for the visits students do to objects already created. The collaboration indicators that can be obtained from active interactions data are the following: (1) *participative*: measures the activity of the learner in the different services, focusing on those contributions that are considered useful; (2) *insightful*: a participative learner that focuses her effort on discussing, commenting, and rating the contributions relevant, to the final solution; (3) *useful*: a participative student that contributes with her comments, ratings, and discussions so that other learners make higher rated new versions of their work; (4) *non-collaborative*: a learner that behaves as if there is no collaboration; (5) *with-initiative*: a learner that starts new activities by her own, and (6) *communicative*: a learner that usually shares information with other learners.

If passive data is used, the following six collaboration indicators can be defined: (1) *thinker-out*: works before doing the contributions, this type of learner reads the survey and the messages related to the learner she is making a contribution to; (2) *unsecure*: goes back to her contributions to confirm that they are correct before making them, thus, before sending her contribution, she reads it several times, and even after having it sent she rereads it again; (3) *gossip*: reads a lot of information without a clear objective, such as surveys, messages, comments, and ratings, but does not produce any contribution related to it; (4) *inspirable*: reads the contributions done by other learners before doing hers; (5) *inspirator*: other learners read this learner's contributions before doing hers; and (6) *thorough*: reads several surveys and messages before rating and commenting the surveys of other learners.

These collaboration indicators can identify different students' performances while interacting in the CLF stages. To detect when a learner is acting as one of the characterized patterns, specific metrics are defined and analyzed during the development of those stages. The aim of these metrics is to obtain as much information as possible on students' behavior independently of the role they are playing (moderator or not). Taking into account the resources provided by the CLF to interact with other colleagues, there are four kinds of metrics: (1) *forum metrics*: related to participation in forums as sending posts, reading answers, navigating through different threads, and so forth, (2) *version metrics*: concern with new versions creation, (3) *rating metrics*: take information from the ratings of students, and (4) *generic metrics*: inform about common website topics as number of connection, hits, or pages visited.

The benefits of using metrics are twofold. On one hand, the information given by the metrics can provide clues to examine the learners' performance, so that instructors can use those parameters to monitor the course. On the other hand, they are the essential elements to make up the collaboration indicators. This is more important from the modeling point of view.

The collaboration indicators definition is the base to conclude if the student's performance suits or not a particular behavior pattern. The student's behavior is being calculated in two ways: (i) using manual rules and (ii) using models based on machine learning algorithms. Manual rules are conditions set for each metric in order to decide when they take a positive value. By default, they follow the definition in [51] but can be changed by the instructor as desired. In turn, supervised machine learning models can be built with data taken from interactions in previous courses and from the current course to infer the users' behavior in an automatic way. In this way, these models can evolve dynamically with their usage and improve their performance and algorithms for next courses. For this, and based on related works and our previous experience [52], decision trees algorithms were used to infer the relationship between the quantitative statistical indicators and student collaboration. These algorithms have been used to analyze student performance [53] because they provided a logical tree that explicitly related performance to quantitative statistical indicators. Decision tree algorithms are a standard technique to classify instances and we did not use other techniques that can offer similar results (e.g., bagging [54]), because the purpose was to obtain metrics with explicit relationships between the dataset statistical indicators and student collaboration features. In this way, the student is classified as whether it belongs to the class corresponding to each indicator or not.

### 3.3. The Adaptation Processes

Besides providing collaboration awareness and motivation through visualizing the collaboration indicators computed for each learner, indicators inferred can also be used to provide adaptive features to the learning environment. The final objective of the collaboration indicators is to support the system adaptation features, so that it can react in different ways depending on learner's behavior. Several adaptation processes can make use of the models inferred, namely, the selection of the moderator, the grouping of the students, and a recommendation process.

The metrics collection represents the part of data mining of the system that is used for the adaptation tasks. All this effort is done with the objective of adapting dynamically the collaboration task to the students' behavior in different directions, on the one hand, to control the collaboration task as defined in the CLF, in particular, the grouping required for the collaboration stage and the moderator selection required in the agreement stage. On the other hand, it provides some recommendations to guide the student to perform specific actions in order to help her with her task, as well as encouraging participation and improving the teamwork. Next, we comment on each of these adaptation processes.

#### 3.3.1. Grouping of Students

After the interaction stage, the system can be asked to group the students considering the collaboration indicators computed. The objective behind this is to produce groups that combine users with different collaboration profiles.

Grouping instances without knowing in advance the most important attributes to organize them can be done by applying unsupervised machine learning techniques such as clustering [55]. In this case we are looking for techniques that are able to discover useful groups that reflect students' behavior. To this end, we followed the available evidence on the use of Expectation-Maximization (EM) algorithm, where the clustering task can be viewed as a maximum likelihood estimation problem and the goal is to find the model structure (number of clusters) that best fits the data [56]. In related research [7], we have already used this algorithm, following examples of other researches, which applied this method in similar circumstances [37, 57]. In this way, the clustering process produces a set of clusters that group learners with similar collaboration indicators. As the aim of this task is to get heterogeneous groups in their collaboration profile, the grouping is done by selecting students from different clusters.

#### 3.3.2. Selecting the Moderator

The choice of the moderator is done according to the students indicators defined during the individual and collaboration stages. The moderator should not be the student with the best valued answer or that who took less time to reach the solution, but the one who has the best skills for communication and the ability to better lead the group discussion. Because of this, when the system chooses the moderator, it takes into account the collaboration indicators computed in the previous stages of the CLF. The moderator is chosen following a priority algorithm. There are two ways to select the moderator by the CLF: through a rule-based approach or through machine learning techniques. Moreover, the human instructor supervising the CLF task can also manually select one of the participants as moderator.

In the rule-based approach, each collaboration indicator has an associated value that represents its influence on the moderator selection. For instance, it can be defined that the most important is “with-initiative,” then “communicative,” then “participative”, and so on. The rule consists in the sum of the indicators representing the performance of every student. In order to select the moderator of the group, the student with the greatest value is chosen.

The machine learning approach is similar to the one used for the inference of the student's indicators. Using classification techniques, a model related to moderator's data can be created using information from former CLF. Using this model and the instances created with the profile of the students in the course, the CLF applies classification algorithms to get those best prepared to act as moderators. The process is as follows. First, the collaboration indicators of each learner are collected and used to build the individual instances. The learned model is used to check if each learner is candidate to become the moderator. If there is more than one candidate, the one chosen is the one who has a higher value in the indicator priorities once they have been added for each candidate. If there is still more than one with the same value, the selection is random.

#### 3.3.3. The Recommendation Process

Depending on the student collaboration profile and behavior, the system can react accordingly by providing personalized individual suggestions. The goal here is to identify recommendation opportunities that guide the student to perform specific actions in the environment in order to help in the collaboration task, encourage participation, and improve teamwork. The purpose here is to associate the recommendations with specific user features (i.e., a collaboration indicator or a set of them) and activate the recommendation when the student behavior suits that pattern. For instance, when the student is identified as “unsecure,” the system could send her a recommendation to spend less time before producing her answer.

In order to define proper recommendations, we have used TORMES methodological approach [58]. It is based on user centered methods to allow the instructors to elicit recommendations that are useful for the learners from a psychoeducational point of view. The idea behind this approach is that recommendations in e-learning scenarios are more complex than recommendations in other domains (such as entertainment) and require the involvement of the instructor in its definition. In that work, 32 educational oriented recommendations that focus on promoting active participation of learners and strengthening the sharing of experience when working in a learning environment were identified from 18 educational scenarios elicited from educators' practice. Although a similar approach should be followed to identify recommendation opportunities for the CLF, some of the recommendations already identified can be considered as a starting point, such as (1) recommending to read a message in the forum contributed by another learner that has keywords that match the learner's interests, (2) recommending to give feedback (rate/comment) on the contributions done by another learner to some previous contributions of the learner recommended, (3) recommending to see the profile of the learner's group mates when the learner is working in a collaborative task and has not read yet their profiles to get to know other classmates, (4) recommending to fill in the information about her profile (photo, webpage, and biography) to a learner who has not done it yet to share personal information for collaboration, and (5) recommending to read the new contributions done by the group mates in a collaborative task that the learner has not read yet. Other recommendations that can be considered are (i) reviewing the answer of a colleague, (ii) creating a new version, and (iii) send messages in the forum, and so forth.

### 3.4. Remarks on the CLF

Our approach for the CLF gathers passive and active data from learners' interaction and uses this information to compute collaboration indicators that can be used to produce an adaptation process that supports learners' collaboration in an intelligent way. Two inference methods are applied to model the collaboration features of the learners: (i) a rule-based approach that follows the proposed definition of the indicators but which can be further modified and (ii) a classification algorithm that learns the model from users' interactions. Several adaptation processes can make use of the models inferred, namely, the selection of the moderator (using classification techniques or a rule-based approach), the grouping of the students (based on clustering techniques), and a recommendation process (delivering recommendations identified with TORMES user centered design methodology).

Furthermore, the CLF is a scrutable tool, and hence, learners can see at any time the indicators computed for them following the open model strategy, which we have successfully applied elsewhere [8]. They are also given access to the metrics used to compute them.

In this way, in our approach for the CLF, we cover the three categories proposed by Soller et al. [6] since this tool not only displays basic actions to collaborators (mirroring system) in terms of sharing proposed solutions and facilitating communication, but also represents the state of interaction via a set of collaboration indicators (metacognitive tools) in a scrutable way and it can offer advice based on an interpretation of those indicators through recommendations (coaching system).

The CLF also addresses aforementioned five conditions [5] as it allows for a shared goal (condition 1), while all participants work together in solving the task, it supports individual accountability in the individual stage (condition 2), it promotes interaction (condition 3) by making participants rate and comment the group mates contributions, everybody works effectively with each other (condition 4) for the same reason as the previous condition, and group functioning is processed and computed with the collaboration indicators provided (condition 5).

## 4. Formative Evaluation

The CLF has been implemented [3] as part of a framework that provides adaptive collaboration support in an open and standards-based learning management system called dotLRN [59]. This approach combines adaptation rules defined in IMS Learning Design specification as suggested in [9] and dynamic support through recommendations via an accessible and adaptive guidance system.

As introduced before, designing the collaboration support is not sufficient and formative evaluations are needed to assess that the system developed truly meets the user requirements. For this reason, we have carried out two observational studies with users, which are reported here. The goal behind them is to improve the design of the CLF and thus enrich the personalized support provided. Since these are formative evaluations, no general conclusions about the approach are expected to be achieved at this stage, but they can provide valuable information to improve the CLF design.

In the first one, we analyze, following the layered evaluation approach, the results of an observational study with 56 participants at the 2009 Madrid Science Week for high-school students (2009 MSW). The second is another observational study with 17 university participants at the 2012 Madrid Science Week for general public (2012 MSW). In them, we analyzed both quantitative data obtained from the logs in the system and qualitative data obtained through questionnaires from participants. Following ethical considerations regarding privacy issues, the analysis of the interaction results was done anonymously, by assigning an identifier to each participant, so interactions and responses to questionnaires were not assigned to the participant's real name.

### 4.1. Observational Study at 2009 MSW

The first version of the CLF has been formatively evaluated in a real scenario during the Week of the Science for high-school students that took place in Madrid organized by the Spanish National Museum of Science and Technology. The goal of this observational study was to understand participants' perceptions on the CLF task and the collaboration indicators proposed.

#### 4.1.1. Settings

56 high-school students (between 14 and 17 years old) were supported in a collaborative session with the CLF. A total of 14 CLF were run. Each CLF activity consisted in a gymkhana where the participants had to write collaboratively a short story taking as input three scientific elements given: a scientist, an invention, and a place. There was one CLF phase, where the 3 stages were offered in a 25-minute period. Participants had to make groups of four in order to participate in the session. Previous to the activity, participants were told about the CLF methodology and shown how the system had to be used, as defined in the interaction stage. The activity was organized as a competition where the group who scored better in the CLF and had a highly rated story was given a prize. The jury was made up by the four aDeNu researchers who were in charge of the study. Moreover, four additional researchers with experience in user studies were also observing the participants during the study and taking notes on the collaboration indicators that they would consider for each participant. This information was very useful to evaluate the system, as commented below.

#### 4.1.2. Study Procedure

The evaluation carried out in this observation study focused on the inference of both the collaboration indicators and the selection of the moderator based on these indicators and the metrics defined for this adaptive process. Details about the evaluation results are provided elsewhere [3]. However, in that paper, the evaluation was not performed following the layered evaluation approach. In order to identify problems in the adaptation process, we have now carried out a layered evaluation. Thus, in order to proceed with the evaluation of the system, we applied the framework proposed by Paramythis et al. [41], which unifies and organizes previous layered approaches into a single framework. The layers defined in this framework are (i) collection of input data, (ii) interpretation of the collected data, (iii) modeling of the current state of the world, (iv) deciding upon adaptation, and (v) applying adaptation. They are complemented with the evaluation of the adaptation as a whole. Next, we comment on the results in each of the corresponding evaluation layers, as well as the evaluation of the adaptation as a whole.

*Layer 1: Collection of Input Data*. The goal is to assure that the data acquisition process is done properly. It relates to both active interactions stored in the learning platform database and processing of the passive data. A testing plan was defined and applied to verify that the data was properly collected. The system was intensively tested during the development phase, using both white and black box tests. All problems detected were solved.

*Layer 2: Interpretation of the Collected Data*. At this stage, we have to validate that the data collected is properly interpreted. During the session, the collaboration indicators were computed using the rule-based approach because, as it was the first running of the CLF, decision tree classification models were not created yet as they depend on learning from past interactions. Therefore, a secondary objective of the study was to get real usage data for learning the classification model that could be used in subsequent experiments. As we were monitoring the indicators being computed along the session, we noticed that the initial value for the rules of numeric metrics (i.e., those that consist of a numeric value such as the number of hits per session) had to be modified because at first most of the computed indicators were out of range.

There are other general problems that may come up from the assumptions made on the user behavior, such as assuming that, when a user is visiting a page, she is actually reading it. Unfortunately with the current settings it is not possible to have certainty that the user has read the page, but it is an educated assumption that is considered in the literature. Enriching the settings with sensors such as eye-trackers and pressure detection on the chair can provide further insight for the real activity of the user, but might turn the session into a more intrusive experience [60]. The second study carried out and reported in Section 4.2 considers the use of sensors.

The approach followed in this first study is to assume that the above interpretation is valid, and, if problems in later layers are detected, this analysis should be reconsidered. These issues are to be detected since the goal of the layered evaluation is to be able to find out where the problem is when the adaptation does not work properly.

*Layer 3: Modeling of the Current State of the World*. This layer focuses on validating the knowledge inferred from the user and checking if the information gathered is reliable to build the classification models. Thereby it deals with the collaboration indicators computed from the data collected and interpreted.

For the testing, two analyses were done. First, students were asked if they agreed with the collaboration indicators computed by the system. Second, the indicators obtained by the system were checked with the indicators written down by the aDeNu researchers who were observing the session.

Results showed that students mostly agreed with the computed indicators. However, there was discord on those aspects focused on indicators with a negative significance (“non-collaborative”: 100% disagreement; “gossip”: 66% disagreement; “unsecure”: 100% disagreement) probably because participants do not accept to be characterized with adverse skills. The other indicators were accepted in more than 80% of cases. As a lesson learnt from this, it is to be researched if the definition of indicators with negative meaning should be displayed in a more positive way.

Regarding aDeNu researches' observations on the indicators, we found some differences between both the observed and the computed ones, especially in those indicators with a behavior highly recognizable from the observation point of view but roughly modeled by the metrics as “with-initiative” (observed 24; computed 3) or “gossip” (observed 27; computed 15). Differences were also found on those elusive indicators with not-so-clear meaning for humans as “insightful,” “thorough,” or “useful.” The system was able to find several samples (12, 6, and 13, resp.) while the observers merely found a few cases (2, 2, and 6).

In turn, the indicator “communicative” seems to have a high level of correspondence (observed 29; computed 24). However, a deeper analysis showed that only 15 of the computed ones matched up to the 29 detected by aDeNu researchers. Therefore there were 9 (24–15) who were identified as “communicative” by the CLF but not so by the instructors and 14 (29–15) who were identified by the instructors but not so by the CLF.

These results are to be considered in the rule-based approach in order to reconfigure the metrics defining those indicators. Likewise, these results can also be used as a better input for the classification process.

*Layer 4: Deciding upon Adaptation*. At this layer, we have to validate if the metrics composition is adequate to the participants interaction and is reasonable to select the moderator. The moderator was selected taking into account the collaboration indicators produced and applying the corresponding predefined rules to select the moderator.

By asking the participants, responses showed that 76% of the participants agreed with the selection of the moderator carried out by the system. Taking into account that the moderator had an extra prize, this result can be considered quite meaningful. From aDeNu researchers' point of view, the selection was also well evaluated.

*Layer 5: Applying Adaptation*. At this layer, the validation deals with how the adaptation was presented to the participants and if they liked it or not. In this study, the adaptation consisted in the moderator selection. So the results from the previous layer apply here too.

*Evaluation of Adaptation as a Whole*. This refers to evaluating the big picture, in other words, if the adaptation offered has facilitated the learning process and the activity performance. In this case, it was obtained that 98% of the participants answered that in their experience the CLF activity promoted the working in the group. By asking the researchers who have been observing the session, all of them agreed with this result (100%).

Moreover, from the observations and the log analysis, it was seen that all the participants worked in a collaborative way, as all of them published their individual solution, rated their group mates, and commented to at least two of the group mates' contributions.

#### 4.1.3. Study Results and Discussion

As a result of this layered analysis, the evaluation shows that the CLF approach allows the creation of effective scenarios that enable learning through interaction, exploration, discussion, and collaborative knowledge construction. The system was able to (1) get metrics from the interaction, (2) build the participants' profiles, (3) identify learners' behavior through the capture of metrics while they are working collaboratively, and (4) establish the base to generate recommendations according to those profiles. Actually, this paves the ground of the adaptation to be provided by the system in terms of recommendations. If we are able to identify how the participants work (and we did, even though some adjustments are needed), then we could guide their interaction in the CLF through recommendations.

Moreover, the CLF provides the appropriate tools to configure the metrics, the rules, and the indicators defining those performances. It also provides scrutability capabilities. This feature allows for a high level of flexibility to the system and can be used to improve the system performance, as commented below.

In relation to the differences found in the third layer (modeling of the current state of the world), we have to consider that collaboration indicators have been computed by the CLF using manual rules defined from the metrics. However, the CLF can also compute the collaboration indicators with classification algorithms from data models (machine learning techniques), but for this latter case it is necessary to have a reliable model to work with (at that moment in time the available models were built from simulated interactions and thus their results underperformed those obtained from manual rules). This is due to the fact that in this study we were using the prototype with real users for the first time. However, the data gathered from this study is to be used to refine the rules design and to build more accurate models for the machine learning algorithms. For this, the scrutability capabilities of the CLF are very useful. In particular, this feature can be used to get labels for the training examples as participants can indicate if they agree with the values computed for the collaboration indicators. This information can be taken into account in the machine learning process and is expected to reduce the differences found in the above evaluation.

We also noticed during the evaluation that the definition of some indicators might have overlapped the description of others. This result is not surprising as the list of indicators offered was tentative, to be further refined after studies with users. This problem was found both during observation and when configuring the metrics of each indicator. An approach to be investigated on this issue is to consider a reduced number of indicators, grouping those with similar meaning (e.g., “thinker-out” and “thorough,” “communicative” and “participative”). Another alternative is to set up a range of possible values for labeling each indicator instead of using their absolute labels. For instance, the system could consider the learners as none, a little, quite, or fully “participative.” Moreover, some indicators should also be redefined in a more positive way. In case of considering that negative values are to be kept, their evaluation could be confirmed by the instructor or by a fellow participant, instead of the own participant.

From the analysis of the results obtained in this first observational study, the focus of the development has to be put on improving the collaboration indicators definition and the data taken from this activity. Further experiments with classification algorithms, following the approach described elsewhere [52], are needed to compute the indicators. Altogether, the data obtained in this study provides a valuable source to be used in improving the machine learning models to be applied in future large scale experiments, as it has been done before [7].

### 4.2. Observational Study at the 2012 MSW

This study was conducted to introduce affective computing in order to improve the design of the CLF. In particular, the goal of this study was to test the infrastructure required to gather emotional information from students. This was meant to get some insight into the potential benefits of considering emotional information sources during the interaction in a collaborative task. In particular, there were two issues of interest. The first issue is extending interaction information to have a clearer picture on participant's behavior within the CLF (to cope with those issues raised in the previous layered evaluation). The second one is testing if the collaboration indicators could be refined in terms of emotional information revealed over time during participants' interaction. Additionally, the perceived usability of the CLF was also measured in the study.

#### 4.2.1. Settings

We prepared four stands in our laboratory that had the infrastructure to gather emotional data from different sensors allowing for physiological recordings (electrocardiogram, galvanic skin response, respiratory rate, and body temperature) and behavioral recordings (face features extraction, keyboard and mouse logs). Moreover, participants were also asked to fill in (1) specific personality traits questionnaires such as the Big Five Inventory (BFI) [61], the General Self-Efficacy Scale (GSE) [62], and the Positive and Negative Affect Schedule (PANAS) [63], (2) subjective reports such as explicit emotional reports written in natural language by the participants and the Self Assessment Manikin (SAM) [43] graphical scale. Additionally, the System Usability Scale (SUS) [64] was also used to gather participants' perception on the CLF usability. Except for the SUS, the infrastructure prepared is similar to the one used in a related individual activity also carried out at the 2012 MSW [65].

A total of 17 participants (including pilots) between 21 and 68 years old and taking courses at university took part in the study. Specifically, 5 different CLF were run. The first three CLFs involved 3 participants each and the last two 4 participants each. The whole session was scheduled in 2 hours, allowing for 40 minutes for the CLF. Previous to the activity, participants were explained the CLF methodology and given a sheet with instructions on how to use the tool. In all CLF runs, participants were asked to solve a brainteaser following the CLF approach. A researcher of aDeNu was assigned to each participant along the study, both to control the sensor information and to observe the participant's interaction. For the later, the screen of the participant was duplicated with the VNC application so it could be seen by the researcher without disturbing the participant. An additional researcher (i.e., general researcher) was in charge of controlling the study as a whole.

#### 4.2.2. Study Procedure

To design this study, we took into account recent works in affective computing that suggest that using a multimodal approach that combines different emotional data sources can improve the results obtained by using a single data source [66]. On-going works from our side are showing results in the same direction [67]. With this background in mind, the script of the interaction was as follows. First, participants were introduced to the activity and asked to fill in the BFI and GSE. Then, physiological sensors were placed on the participant, at the same time that the recording of the behavioral sensors was launched. Then, the participant's baseline for the physiological measures was computed. A polygraph-style test was also used to identify participant's individual reactions to physiological signals.

After that, the emotions reaction calibration was done with the SAM scale. Here, 8 images (emotionally standardized) from the IAPS database [68] were selected with decreasing levels of valence and increasing levels of arousal. The purpose was twofold: on the one hand, training the learners with the SAM scale and confirming that the learners have understood its usage, on the other hand, getting participants' baseline emotional values in terms of their valence and arousal.

At this point, participants were asked to use the CLF to solve the requested brainteaser. They were also asked to fill in the SAM after finishing each of the CLF stages (individual, collaborative, and agreement). They also had to report on their feelings typing in a text field where the following sentences were started: (1) “When doing this task, I've felt…”; (2) “When doing this task, I've thought…”; (3) “Difficulties encountered when solving this task have been…”; and (4) “To overcome these difficulties I have…”.

Regarding the CLF performance, the collaboration indicators were computed with the machine learning algorithms trained with the data from the previous study. However, the moderator was selected manually by the general researcher from her observations on participants' interactions. The reason for this was to confirm that selected moderators were those participants more engaged in the activity, along with those who have best skills for communication and the ability to better lead the group discussion.

After the CLF, participants were asked to propose another brainteaser. Due to activity time constraints, for this task the CLF was not used. In turn, participants were only asked to sketch their proposal individually. However, it would have been of interest to run a second CLF with the same participants for this latter task.

When finished and after computing the final baseline for the physiological signals, participants' sensors were removed. Then, participants were asked to fill in the PANAS and SUS questionnaires. After that, they were explained the goals of the aDeNu research in general and the particularities of the study carried out. Time was also allowed for participants debriefing their experience in an informal manner.

#### 4.2.3. Study Results and Discussion

Due to the exploratory nature of the study and the reduced number of participants, results cannot be concluding, but they can provide informative guidance to extend the CLF design with emotional information. Moreover, the first two CLF of the five carried out have not been used in this analysis as they were initial pilots for testing the settings, and thus, lack of information gathering occurred. As a result, the outcomes from 11 participants were analyzed.

**Figure 2:**
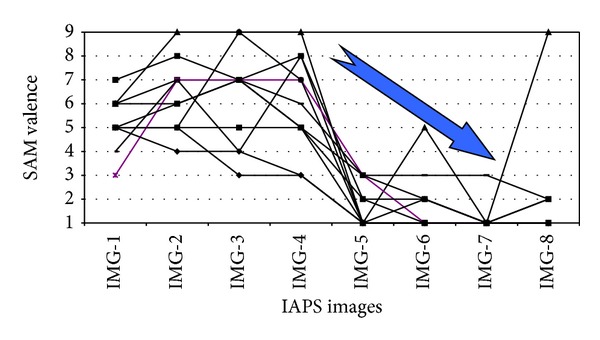
Valence assigned by participants to IAPS selected images.

**Figure 3:**
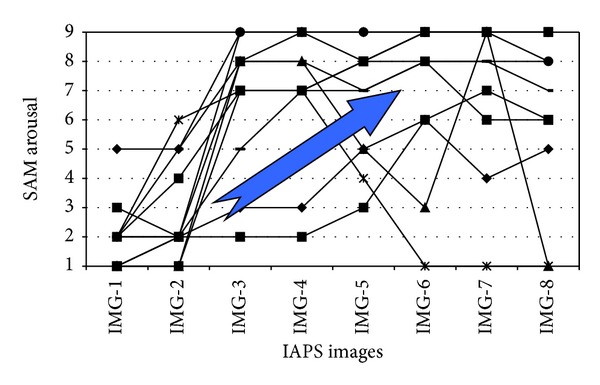
Arousal assigned by participants to IAPS selected images.

First, we comment on the SAM findings. Figures 2 and 3 show the SAM calibration outcomes on the IAPS selected images (valence and arousal values, resp.). It can be seen that all the 11 participants that have been analyzed (except one of them in the valence and two in the arousal) seem to have understood the scale and properly assigned values to it as the graphics show that scores follow the expected trend: decreasing levels of valence and increasing levels of arousal.

**Figure 4:**
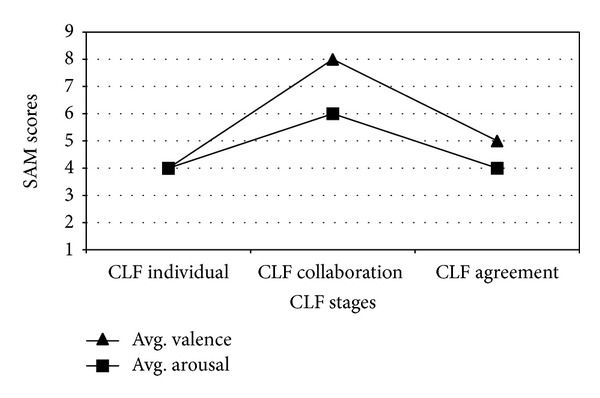
Computed average SAM valence and arousal during CLF interaction stages.

Having this in mind, we computed participants' valence and arousal after each of the CLF stages. Due to operational issues, SAM results were only obtained in one of the CLF for the non-moderators. However, in our view these results could be representative of the behavior in the other CLF executions. Average values obtained are shown in Figure 4.

From the graph in Figure 4, it can be seen that for the non-moderators the collaboration stage of the corresponding CLF seems to be the most attractive (higher valence) and dynamic (higher arousal), while the agreement stage is not perceived as well. In our view, this can be caused by the design of the activity. Since participants were altogether at the same time doing the task, when getting to the agreement stage, non-moderators had to wait till moderators proposed the joint solution.

Regarding the usability perception, results suggest that there are issues to be improved, since results obtained from the 11 participants of the three CLF analyzed were rather low (average: 56.43 out of 100; standard deviation: 13.45). In fact, an average rating of 68 has been abstracted from over 500 studies [69], which means that a SUS score under 68 (like in this case) is considered under average. The most critical usability issues that require improvements are to reduce the steps to carry out certain actions and to make clearer to the participant how to access peer contributions in the collaboration and agreement stages.

Regarding collaboration indicators, a first attempt was done to analyze them with respect to the personality traits gathered from the 11 participants, which are reported in Table 1. The participants' sample seems to be rather homogenous both in the BFI and EAG (standard deviations under 19%).

Regarding the outcomes from the PANAS, it seems that participants had different perceptions after the performance of the CLF task since the range of values for the PANAS is wider.

The selected moderators were the ones that had scored higher in the BFI dimensions except for the openness dimension, where they were in the lower band. It has to be remarked here that moderators' selections were done by the general researcher based on the behavior observed during the individual and collaboration stages, but she did not take into account participants' personality traits or their collaboration indicators computed.

The most computed collaboration indicators (using the machine learning models trained with data from the previous study) were “intuitive” (8 participants), “useful” (8 participants), and “participative” (6 participants). Moreover, apart from these three indicators, selected moderators by the general researcher were classified also by the following indicators: “inspirator” and “thinker-out.”

The above results suggest that the relationship between the personality traits, the CLF roles, and the collaboration indicators might be worth investigating. This is in line with a related approach described elsewhere [49].

At this stage we cannot comment on the findings from the other emotional information sources gathered in the collaborative tasks (i.e., physiological and behavioral recordings) as we are still processing them (several open issues exist regarding the extraction of emotional indicators from the signals gathered). To address some of these open issues, we are designing activities where the specific emotions that we want to identify are forced in the participants in very specific moments during the tasks. We expect that this will allow us to get a better insight on how to process these signals and advance the state of the art in this issue.

With regard to the current findings from this observational study, we have focused the analysis on those sources that can be considered in online settings, as we are designing the next study in this context (see below). However, it can be pointed out that sensors information can allow to get certainty of the participants actual performance on the task and thus help to better interpret collected data when doing a layered evaluation of the adaptation process. For instance, combining mouse and keyboard tracking with eye movements on the screen can inform, with low intrusion level, if the participant is actually working on the CLF.

In summary, in this second study we have identified usability issues that should be addressed to improve the participants' perception of the CLF. We have also identified the need to research the relationship between the personality traits, the CLF roles, and the collaboration indicators. Regarding the infrastructure for collecting data in a collaborative setting, low intrusion and costless approaches are to be used in online collaboration settings.

## 5. Overall Discussion and Future Work

In the previous section we have reported two formative evaluations carried out to improve the CLF design and thus to enrich the personalized support provided by this collaborative tool. Since these are formative evaluations, no conclusive outcomes were expected at this stage, but rather design informative hints were obtained.

From the layered analysis done on the first study, we identified the need to get some certainty on whether the learner is really doing the task when the information is displayed. This can be achieved by using sensors, as suggested in the second study. Moreover, the results analysis of the first study also showed that some indicators were not well understood or computed and it was suggested to consider labeling indicators using a range of values instead of absolute identification. From the outcomes of the second study, we propose to investigate the relationship between the personality traits, the CLF roles, and the collaboration indicators. Moreover, as a result of the second study, some usability issues were identified (i.e., reduce unnecessary navigation steps and present information more clearly). These issues are being addressed by usability specialists of TEC Digital (the Information and Technology Department of the Technological Institute of Costa Rica, TEC), who are currently collaborating with aDeNu Research group.

Another matter is that these two formative evaluations were carried out in a face-to-face environment in order to be able to observe participants' interactions. However, in our view, the CLF gets its higher potential in online settings, with activities that would require several weeks of work, and thus the unpleasant waiting for the moderator proposal in the agreement stage can be removed. In this line, we are preparing a new formative evaluation for the forthcoming 2013 Madrid Science Week. The preliminary study design is as follows. Participants are to be asked to solve 2 brainteasers and propose another one using the CLF approach online. The activity will be open during all the available period and participants will work asynchronously and online. Personality traits will be computed in the same way as in the second study. Regarding the emotional information gathering, our proposal is to compute SAM valence and arousal not only at the end of each CLF stage but also every time the participant logs in to work in the CLF and, if possible, every time she logs out. We are aware that the latter has difficulties, as most probably participants would stop working in the CLF without reporting this event. Sensors could also be used for this detection, but we are not considering them here for the nature of this study (i.e., the activity is to be carried out by any Madrid citizen from home), but they will be introduced in future studies.

By delivering the activity in an online setting, we expect to get a large number of participants and thus to do some statistical analysis regarding the relationship of the personality traits and the collaboration indicators and roles.

Additional future work is the integration of the CLF approach into other e-learning platforms, such as Moodle or Sakai. For this, we are working on the implementation of the CLF as a web service, instead of a packed solution for dotLRN.

## 6. Conclusion

We have presented the CLF approach that supports the creation of effective scenarios that enable learning through interaction, exploration, discussion, and collaborative knowledge construction. Following the CLF, the collaboration modeling considers the context (explaining learners' potential and capacity to collaborate), process (monitoring learners' interactions), and assessment (supporting learners' awareness of own behavior as well as fellows' behavior). These issues are further discussed elsewhere [49].

An intelligent support, which is based on rules and machine learning techniques (i.e., classification and clustering), facilitates its management during the design, conduction, and analysis of the collaborative learning experience and supports both students and instructors. The participation of the students is fostered in the process (e.g., rating capabilities and comments make learners share their opinion on other learners' contributions) and supported in the authoring of their own contributions in a collaborative way. Moreover, recommendations can be provided to guide the interactions in the CLF. At the same time, the approach makes that the instructors' workload is reduced as some of their tasks—especially those related to the monitoring of the students behavior—are automated. The system also provides scrutability capabilities as the collaboration indicators inferred are shown to the user, both student and instructor.

In our view, this approach can help the control and management of the collaboration. Metrics, collaboration indicators, machine learning, and rules are a good base to identify the learners' behavior (as we have verified elsewhere [8]). After discussing the findings in them, an online large-scale experiment is being designed for the forthcoming 2013 Madrid Science Week. Since a large participation is expected, we aim to research with statistical significance (1) how accurate the collaboration indicators definitions and the metric currently considered in the system are and (2) in what way they can be enriched if personality information and emotions are considered. For this, we will take into account the affective collaborative learning modeling depicted elsewhere [49]. This modeling takes into account the analysis of the affective reactions elicited during the collaboration process within the ongoing collaboration task itself and those due to the interaction with peers that feed the collaboration assessment. Moreover, collaboration roles (either scripted such as the moderator CLF or naturally emerged such as social leadership) can elicit an additional emotional reaction or modulate existing ones.

In this line, the second observation study has shed some light on the relation of the collaboration indicators with affective issues. We are currently extending the CLF collaboration model with emotional indicators and personality traits following a domain-independent data mining approach used in previous collaboration studies. The purpose here is to use collaboration indicators to support the system adaptation features, so that it can react in different ways depending on learner's behavior. For this, we need to process all emotional information sources gathered in the study (i.e., physiological and behavioral recordings).

All sources of emotional information deserve future analyses in order to refine and calibrate the affective influence on the collaboration indicators. This has to be articulated using a data mining approach [67]. In this way, potential situations where the CLF collaboration process is interfered and therefore needs to be reoriented can be identified. By introducing the aforementioned affective issues, the approach is expected to improve collaborative learning. In particular, based on our experience in developing educational recommender systems those improved indicators will serve to develop affective educational recommendations [70].

We expect that this paper will motivate researchers of collaborative tools to carry out formative evaluations with users in order to improve the design of their tools and hence enrich the personalized collaborative support provided to their users in the same way we are improving the design of our domain-independent tool for intelligent collaboration support (i.e., the CLF).

## Figures and Tables

**Table 1:** Average and standard deviation for personality traits computed.

	Average	Stand. dev.
BFI-openness	25.22	4.58
BFI-conscientiousness	36.00	2.65
BFI-extraversion	32.89	5.46
BFI-agreeableness	18.33	3.46
BFI-neuroticism	40.44	4.90
GSE	38.33	4.92
PANAS-positive	29.22	7.82
PANAS-negative	16.00	6.63
PANAS-balance	13.22	7.53
